# The auditory stimulus facilitates memory guidance in distractor suppression in males with substance use disorder

**DOI:** 10.3389/fpsyg.2024.1417557

**Published:** 2024-07-03

**Authors:** Biye Cai, Jinjin Wang, Hanbin Sang, Zonghao Zhang, Aijun Wang

**Affiliations:** ^1^Department of Psychology, Research Center for Psychology and Behavioral Sciences, Soochow University, Suzhou, China; ^2^School of Physical Education and Sports Science, Soochow University, Suzhou, China; ^3^Department of Sports, Kangda College of Nanjing Medical University, Lianyungang, China; ^4^Key Laboratory of Child Cognition and Behavior Development of Hainan Province, Haikou, China; ^5^School of Teacher Education, Qiongtai Normal University, Haikou, China

**Keywords:** substance use disorder, audiovisual enhancement, cognitive control, working memory, attentional guidance

## Abstract

**Introduction:**

Representations in working memory can affect distractor suppression in human visual search, and this process is modulated by a separate top-down cognitive control. An increasing body of research has demonstrated that patients with substance use disorder (SUD) have deficits in cognitive control over filtering interference by perceptual distractors. However, their ability to resist proactive interference from working memory has received comparatively less attention.

**Methods:**

Here, we investigate this issue by employing a working memory/visual search dual-task paradigm. An intervening gap-location search task was instructed to be performed while participants memorized a written color word, with congruent auditory information present during the memory encoding phase on half of the trials.

**Results:**

Results showed that there was a reliable response time (RT) advantage when the meaning of the memory sample agreed with the color of one of the distractors under the visual alone condition. However, such a result was only found in the control group. More importantly, both groups exhibited comparable facilitation under the audiovisual condition, with the facilitation effect appearing later in the SUD group. Furthermore, the facilitation effect was superior in magnitude and time course under the audiovisual condition to the visual alone condition.

**Discussion:**

These findings highlight how patients with SUD resist distractor interference at the memory level and extend our understanding of how working memory, selective attention, and audiovisual enhancement interact to optimize perceptual decisions in patients with SUD.

## Introduction

Substance use disorder (SUD) is widely recognized as a common mental illness, and encompasses an ongoing pattern of substance seeking and use despite adverse consequences for an individual’s psychology and physiology (Kessler et al., 2005; Zilverstand et al., 2018). Many studies have demonstrated that SUD is linked to a range of cognitive dysfunctions (Smith et al., 2014; Ceceli et al., 2023). Typically observed impairments include deficiencies in selective attention, inhibitory control, working memory, and decision-making (Potvin et al., 2014; Verdejo-Garcia et al., 2018). Within the scope of cognitive function, cognitive control is most apparently impaired in patients with SUD (Goschke, 2014; Hildebrandt et al., 2021), a resource-demanding system that ensures the achievement of task goals by suppressing inappropriate or irrelevant immediate responses (MacDonald et al., 2000). In the fifth edition of the Diagnostic and Statistical Manual of Mental Disorders (DSM-5), four out of the eleven symptom criteria on which the diagnosis of SUD is based are categorized as impaired cognitive control, underlining the relevance of this process for SUD (American Psychiatric Association, 2013).

Ample evidence has been found that cognitive control impairment in patients with SUD (Luijten et al., 2014; Zhukovsky et al., 2022). A meta-analytic study indicated that patients with SUD manifested deficits in behavioral and neuropsychological tasks measuring inhibitory control and working memory (Garavan and Hester, 2007), especially on tasks requiring high demands for cognitive control (Kübler et al., 2005). For instance, using a Stroop color-naming task, Battisti et al. (2010) found that cannabis users showed increased errors and RTs during color-incongruent trials compared to non-using controls, and that their poorer performance was associated with earlier onset of regular cannabis use. Recently, research by Ruiz et al. (2019) reported a greater semantic interference effect in chronic cocaine users and recreational cocaine polydrug users than normal controls. Furthermore, fMRI evidence has revealed that substance users show decreased recruitment of executive networks during cognitive control tasks, a pattern that is similar across people who use different types of substances (Feil et al., 2010; Zilverstand et al., 2018). Together, the above findings meant that SUD was intimately related to an overall deficit in top-down cognitive control.

Recent research has provided evidence that cognitive control can not only play a major part in filtering distractor interference in visual perceptual and attentional tasks, but also influences another vital capacity for inhibition function, namely resistance to proactive interference at the memory level (Han and Kim, 2009; Zhang et al., 2020; Cai et al., 2024a,b). According to the biased-competition theory, when a set of objects is kept in working memory, attention is biased toward matching items over other competing stimuli in a top-down way (Desimone and Duncan, 1995). Numerous studies have provided evidence for this theory by demonstrating that perceptual stimuli that match representations held in working memory can attract attention even if they interfere with the immediate task (Soto et al., 2005; Olivers, 2009; Sasin et al., 2022). Soto et al. (2005), for instance, employed a working memory/visual search dual-task paradigm and discovered that responses were slower in conditions where memory items were color-matched to the distractor than in color-mismatched conditions, suggesting attentional guidance by working memory. This phenomenon was initially recognized as an automated, uncontrolled process (Olivers et al., 2006).

Nonetheless, some studies have found that memory-matching distractors do not always automatically capture attention and can even be effectively suppressed through top-down cognitive control mechanisms (Carlisle and Woodman, 2011a; Wen et al., 2018). Attentional capture effects by memory-matching distractors were reversed into the attentional suppression effect if participants were told in the experimental instructions that the memorandum would never share the same properties with the target in the search display (Woodman and Luck, 2007). That is, people could control the allocation of attention to avoid interference by a memory-matching distractor, thereby optimizing the visual search (Han and Kim, 2009). Furthermore, several studies have found individual differences in memory-driven attentional capture, as evidenced by the fact that this effect is modulated by an individual’s cognitive control ability (Cai et al., 2022). For instance, focusing on a relationship between anxiety and memory-driven attentional capture, Luo Y et al. (2021) demonstrated that high-anxiety individuals were more vulnerable to memory-matching distractors than low-anxiety individuals because anxiety had a negative impact on the cognitive control system. Combined with the aforementioned findings, we would expect that people with SUD have a weaker cognitive control ability to effectively utilize working memory representations to guide attention and then resist distractor interference from working memory.

Meanwhile, evidence has shown that congruent auditory stimuli presented in synchronization with visual working memory content contribute to enhanced cognitive control, which in turn modulates attentional allocation and improves search efficiency (Cai et al., 2024a,b). Studies conducted on children with attention-deficit/hyperactivity disorder examined the influence of congruent audiovisual memory encoding on working memory guidance by manipulating memory modality in a working memory/attention dual-task paradigm (Cai et al., 2022). It was discovered that attentional capture by memory-matching distractors was eliminated by congruent audiovisual maintenance. The authors attribute this phenomenon to an interaction between audiovisual memory encoding and cognitive control, which creates a robust rejection template and thus facilitates attentional suppression. Moreover, Cai et al. (2024a,b) used a cumulative reaction time (RT) distribution method to further discover that congruent auditory memory content not only facilitated the effect size of memory-guided distractor suppression effect but also facilitated its time course, leading to a stronger suppression effect and an earlier onset. Thus, the present study sought to explore whether the congruent auditory memory content could restore cognitive control and thus facilitate memory-driven attention in strength and temporal dynamics in individuals with SUD. Previous studies have shown that the prevalence of illicit drug use is significantly higher among men than women (Center for Behavioral Health Statistics and Quality (CBHSQ), 2016; United Nations Office on Drugs and Crime, 2023), although this sex gap is shrinking rapidly and that compulsory isolated detoxification is the primary treatment model for people who use drugs currently practiced in China (Yang and Giummarra, 2021). Therefore, studying the resistance to proactive interference from working memory of male compulsory detoxification, as well as the ways to improve it, has a wide range of representativeness and important application value for developing targeted interventions to prevent relapse (Lu et al., 2024).

In the present study, we aimed to investigate these two issues by employing a working memory/visual search dual-task paradigm, in which a congruent auditory stimulus was presented in synchronization with visual working memory items on half of the trials. Given that previous studies have demonstrated abnormal cognitive control in patients with SUD (Garavan and Hester, 2007) and the auditory facilitation effect of cognitive control over working memory guidance (Cai et al., 2024a,b), we predicted that an evident memory-guided distractor suppression effect was observed across percentiles of the RT distribution under the visual alone condition in the control group, whereas such an effect was absent in the SUD group. On the other hand, both groups exhibited a comparable suppression effect under the audiovisual condition, with the later onset of the suppression effect in the SUD group compared to the control group. Additionally, we aimed to investigate the association between working memory guidance and self-report measures of traits related to substance addiction (i.e., self-control, anxiety, and relapse tendency) in an exploratory fashion. Considering the relationship between memory-driven attention and cognitive control (Luo Y et al., 2021) and between cognitive control and substance addiction (Zilverstand et al., 2018), we anticipated that smaller memory-guided distractor suppression would be related to stronger substance addiction.

## Methods

### Participants

A prior power analysis was conducted to estimate the appropriate sample size for a three-way repeated measures analysis of variance (ANOVA). With projected η_p_^2^ set to 0.25 and α err prob. set to 0.05, 46 participants were required to achieve 90% power according to G*Power 3.1 (Faul et al., 2007). Fifty eligible male adults were enrolled in the study, including twenty-five individuals with SUD (mean age: 31.80 ± 4.90 years) and twenty-five health controls (mean age: 30.92 ± 4.43 years). Individuals with SUD were recruited from the Taihu Compulsory Rehabilitation Centre, Jiangsu Province, China, and met specific inclusion criteria: (1) 18–40 years old, (2) current DSM-5 SUD diagnosis, (3) at least 30 days of abstinence from any drug, (4) right-handed, (5) normal eyesight or corrected eyesight and normal auditory acuity, and (6) no serious neurological disease. Twenty-five demographically-matched health control participants were recruited by convenience, by snowball communication, from the local community, and all of whom met inclusion criteria: (1) 18–40 years old, (2) no history of illicit drug use, (3) no use of alcohol and tobacco in the last 30 days, (4) right-handed, (5) normal eyesight or corrected eyesight and normal auditory acuity, and (6) no serious neurological disease. Each participant gave informed consent before the research, in compliance with the Declaration of Helsinki. This research was granted by the Academic Committee of the Department of Psychology, Soochow University.

### Materials

#### Basic information

Participants’ drug abuse history and demographic characteristics were collected and listed in Supplementary Table S1. Demographic characteristics included age, educational background, career, marital and family status, and children’s status. Drug abuse history included type of drug use, number of compulsory detoxifications, and years of drug use. There were no significant differences in demographic characteristics between the SUD group and the control group, *ps* > 0.19.

#### Relapse Tendency Questionnaire

The Relapse Tendency Questionnaire (RTQ) was employed to assess the probability that participants will resume drug use after finishing treatment (Zhu and Geng, 2002). The RTQ is a five-dimensional scale, with a total of 18 items. It assesses willingness to quit drugs (my resolve not to relapse into drug use), substance substitution (the frequency with which scenes related to past drug use come to my mind), objective environment (difficulty in obtaining drugs after detoxification), physical and mental condition (the effect of chronic drug use on my spirit and willpower), and social support (the extent to which family, friends, and relatives supported and encouraged me to quit drugs). A 6-point Likert scale was utilized with a range from 0 (the lowest level of severity) to 5 (the highest level of severity), where higher overall scores suggest a stronger tendency toward relapse. The scale has good reliability and internal consistency (Sun et al., 2023; Zeng and Wei, 2023). In this study, Cronbach’s alpha coefficient was 0.83, showing good internal consistency.

#### Brief Self-Control Scale

The Self-Control Scale (SCS), comprising 36 items, was initially introduced by Tangney et al. (2004) and has been widely accepted as a robust instrument for assessing an individual’s overall ability to manage impulsive reactions. Subsequently, Morean et al. (2014) developed a simplified version, the Brief SCS, consisting of 7 items delineating the dimensions of impulse control (4 items) and restraint (3 items). Participants evaluated the extent to which each statement resonated with their experiences using a 5-point Likert scale (1 = Not at all like me, 5 = Very much like me). For instance, an exemplar item was “I can resist temptation well.” The cumulative score across the 7 items represented one’s self-control capacity, with a range from 7 to 35, where higher scores correspond to higher levels of self-control (Lindner et al., 2015). In this investigation, the Chinese version of BSCS, as developed by Luo T et al. (2021), was utilized to assess the level of self-control. The Chinese version of the scale has been shown to have good validity and reliability (Luo T et al., 2021; Zhang et al., 2022). The internal consistency of the present data was adequate, as reflected by a Cronbach’s alpha coefficient of 0.85.

#### State–Trait Anxiety Inventory

The State–Trait Anxiety Inventory (STAI) was a self-report rating inventory that presented 40 items related to personal feelings (e.g., happy, nervous, and secure), with half of the items measuring trait anxiety and the rest measuring state anxiety. That presented 40 items related to personal feelings (e.g., happy, nervous, and secure), with half of the items measuring trait anxiety and the rest measuring state anxiety (Spielberger, 1983). The trait anxiety subscale measures relatively stable long-term anxiety, whereas the state anxiety subscale examines short-term state anxiety in a specific situation. Participants rated the appropriateness of each statement on a 4-point Likert scale, ranging from 1 (seldom) to 4 (almost always). The score range for each subscale (i.e., state and trait anxiety subscale) is 20–80, where elevated scores denote increased levels of anxiety. The Chinese version of STAI developed by Shek (1993) and Shek (1988), which has good internal consistency and reliability (Ma et al., 2013), was adopted in the present study. For both the trait and state anxiety subscales, Cronbach’s alpha coefficients were 0.90, respectively, indicating good internal consistency.

#### Stimuli and apparatus

The experiment task was administered using “E-prime” software (version 3.0). The visual stimuli were displayed on a 16-in. LCD screen (resolution 1,024 × 768, refresh rate 60 Hz), and the screen background color was kept gray (RGB: 128, 128, 128) during the experiment. The auditory stimuli were recorded by a female Chinese naïve speaker and delivered at standardized amplitude via stereo headphones (model: HyperX). During the experiment, participants sat about 57 cm in front of the monitor.

The experiment was composed of two different memory stimulus presentations: visual alone: the memory items were five written color words (1.29° × 1.29°) printed in white, which included “红,” “绿,” “蓝,” “棕,” “紫” (corresponding to red, green, blue, brown, and purple in English, respectively); audiovisual combinations: the memory items were combinations of the above five Chinese characters and their spoken sounds [e.g., the spoken sound “\hong\,” corresponding to red (\rεd\) in English, was paired with the written color word “红”]. The memory probe was five written color words (1.29° × 1.29°) randomly selected from the same stimulus pool as the memory sample. Four colored squares (2.5° × 2.5°) were positioned at four locations (2, 5, 8, 11 or 1, 4, 7, 10 o’clock with equal possibility) around an imaginary circle centered on the fixation (radius 6.5°) for the search task. The thickness of the border line of the colored square was 0.07° in visual angle. One target with a slighter larger gap (0.07° × 0.43°) on the left or right side was displayed in three distractors with equal-sized gaps (0.07° × 0.29°) on both sides. The target locations and orientations occurred with equal probability and were administered unpredictably. The colors of the squares were drawn randomly from a pool of five equiluminant (13.1 cd/m^2^) colors [in CIE coordinates: red (0.70/0.33), green (0.22/0.77), blue (0.16/0.09), brown (0.57/0.45), and purple (0.37/0.27)], and the color of each search item in the search display was unique. The cue stimuli agreed with the search items with the exception that there were no gaps on either side.

#### Procedure

Each participant first underwent a memory-driven attention task and then answered four questionnaires in paper form and a fixed order: Basic Information Questionnaire, RTQ (Zhu and Geng, 2002), BSCS (Luo T et al., 2021), and STAI (Shek, 1988).

**Figure 1:**
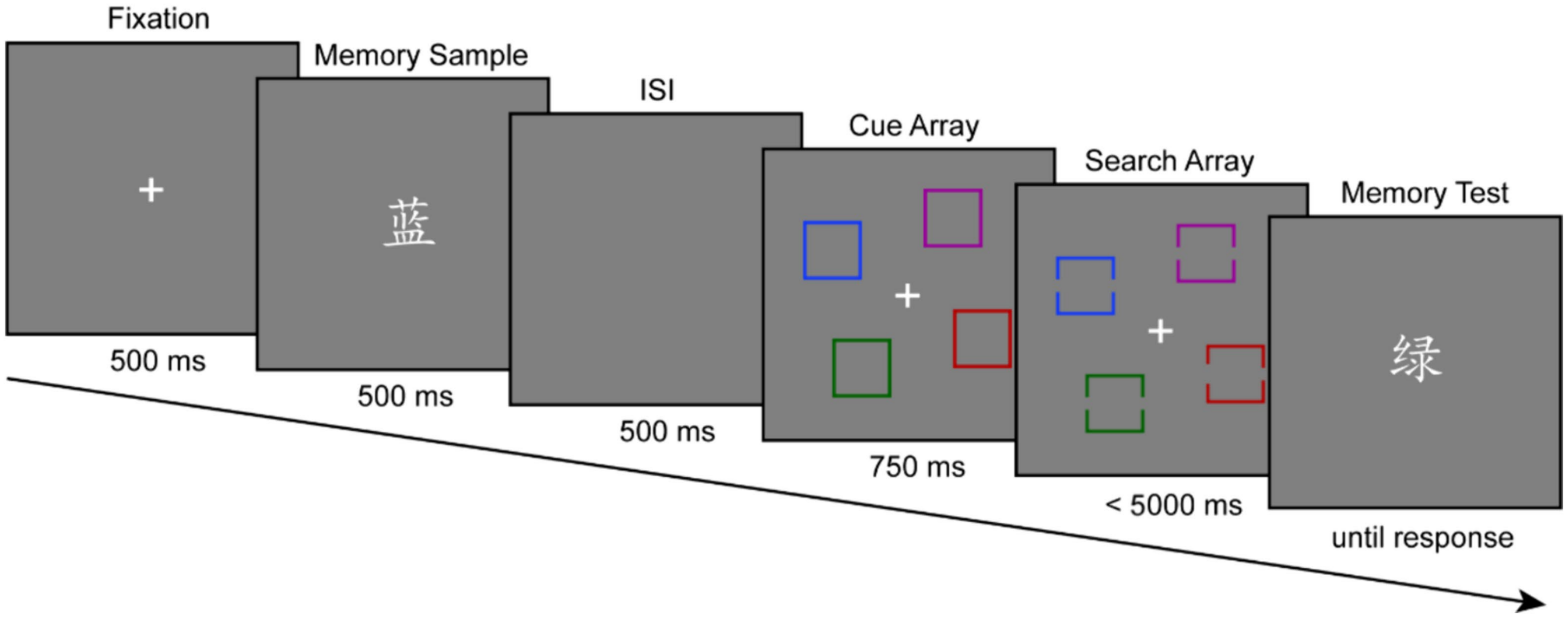
A example of a schematic illustration of the sequence of events in the experimental procedure. Participants were asked to remember a memory sample delivered via either visual or audiovisual modalities (e.g., [蓝 (\lan\), corresponding to blue (\blu\) in English]) for a later memory test. During memory retention, participants were required to find a colored square with a slightly larger gap on either side (target) from three colored squares with equal-size gaps on both sides (distractor) and report on which side of the larger gap of the target was located. One of the distractors could be a match or a non-match to the color described by the memory sample. Participants were then given a memory test probe (e.g., [绿 (\lv\), corresponding to green (\grin\) in English]) and asked to report whether the probe word matched the initial memory sample. The cue array was presented between the memory display and the search display, providing prior knowledge of the color and location of search items.

For the memory-driven attention task, a dual-task paradigm was used in this experiment that consisted of a delayed match-to-sample working memory test, interleaved with a gap-location search task during the delay. (see Figure 1). The first 500 ms of every trial began with the presentation of a white central fixation cross (1.5° × 1.5°). A written color word was then displayed alone in the center of the screen (visual alone) or accompanied by its spoken sound (audiovisual combinations) for 500 ms (memory sample). Participants were required to memorize the stimulus for a later memory test.

A cue array consisting of four colored squares showed for 750 ms following a 500 ms delay interval, giving *a priori* information about the color and placement of the next search item. When cue items were displayed, participants were only asked to look at the cue stimuli without making a button response. Immediately thereafter, the search target, an outlined colored square with a slightly larger gap on either side was presented on the screen simultaneously with three distractors, outlined colored squares with equal-size gaps on both sides, with a maximum presentation time of 3,000 ms (search array). Participants had to identify which side of the target square the slighter larger gap is located by pressing a button as fast and precisely as they could. If the larger gap was positioned to the right side of the target square, participants had to press the “J” key, and vice versa with the “F” key (50% each of the total trials). There were two types of trials, distinguished by the association between the memory sample and the search array. On distractor-match trials, the meaning of the memory sample agreed with the color of one of the distractors in the search display. On no-match trials, the meaning of the memory sample differed from the color of all search items. Two types of trials happened in randomized order and with equal frequency.

Next, a written color word was displayed at the centre of the screen (memory probe) and remained visible until the participant had responded. Participants were required to indicate if the memory probe matched (“1”) or did not match (“2”) the memory sample as accurately as possible without time pressure. On half of the trials, the memory sample and the memory probe were the same, while on the other half, they were different. Feedback was provided to participants for every search array and memory probe. An inter-trial interval averaging 1,200 ms (jittered between 1,000 ms and 1,500 ms) separated each trial. Participants were instructed that the color and placement of the cue stimuli agreed with that of the search items and that the meaning of the memory sample must be different from the color of the search target. Previous research with healthy adults has shown that feature-based ignore cues (color and location) are a critical experimental setup for inducing memory-guided distractor suppression (Han and Kim, 2009; Moher and Egeth, 2012; Cai et al., 2024a,b).

The experiment was designed as a 2 (stimuli presentation type: visual alone, audiovisual) × 2 (matching type: distractor-match, no-match) × 2 (group: SUD, control) mixed experiment. To get familiar with the task, participants were required to complete a practice block (24 trials), followed by 128 trials spread across 4 blocks of 32 trials. The matching type was randomly intermixed within each block. For each participant, the same stimuli presentation type was presented in two consecutive blocks. The order of presentation of the stimuli presentation type followed an ABBA design and was counterbalanced across participants. The whole experiment took approximately 25 min.

### Data analysis

Before analyzing the search RTs, trials with incorrect responses in either the search or the memory task and trials with no responses in the search task were excluded, as well as RTs greater than ±2.5 SDs of the mean search RT for each participant within each condition. These criteria excluded 7.66 and 7.47% of trials for the SUD and the control group, respectively. Supplementary Table S2 displays the mean correct rates (CRs) for the delayed match-to-sample working memory test, as well as the CRs and response times (RTs) for the search task. SPSS 27 was used to process the data.

We analyzed the data as follows: (i) to investigate whether there were differences in memory and search performance between individuals with and without SUD, a 2 (stimuli presentation type: visual alone, audiovisual) × 2 (matching type: distractor-match, no-match) × 2 (group: SUD, control) repeated-measures ANOVA was applied to mean search RTs, memory CRs, and search CRs, respectively. (ii) Then, independent samples *t*-tests were conducted to examine the differences in the magnitude of working memory guidance (RT_no-match_ – RT_distractor-match_) between the SUD and the control group under two stimuli presentation types. (iii) In addition, we conducted paired sample *t*-tests to investigate how stimuli presentation type affects the magnitude of attentional guidance in both groups. (iv) On the other hand, we calculated and compared the search RT between no-match and distractor-match conditions across conditions using the cumulative probability RT distribution method (Carlisle and Woodman, 2011b; Cai et al., 2024a,b). (v) Finally, we performed Person’s correlations to explore the association between self-report scores on the three scales (i.e., RTQ, BSCS, and STAI) and working memory guidance in each condition.

## Results

### Memory and search correct rate

All participants performed well on both memory and search tasks, with over 95% CRs. For the memory CRs, a 2 (stimuli presentation type: visual alone, audiovisual) × 2 (matching type: distractor-match, no-match) × 2 (group: SUD, control) repeated-measures ANOVA was performed, and revealed a significant main effect of stimuli presentation type, *F*(1, 24) = 23.24, *p* < 0.001, η_p_^2^ = 0.33, indicating that the memory CRs for the audiovisual condition (98.6%) were higher than those for the visual alone condition (95.6%). All other effects were not significant, *Fs* < 1. For the search CRs, a similar three-way repeated-measures ANOVA did not produce any significant effects, *p*s > 0.05.

### Search response time

**Figure 2:**
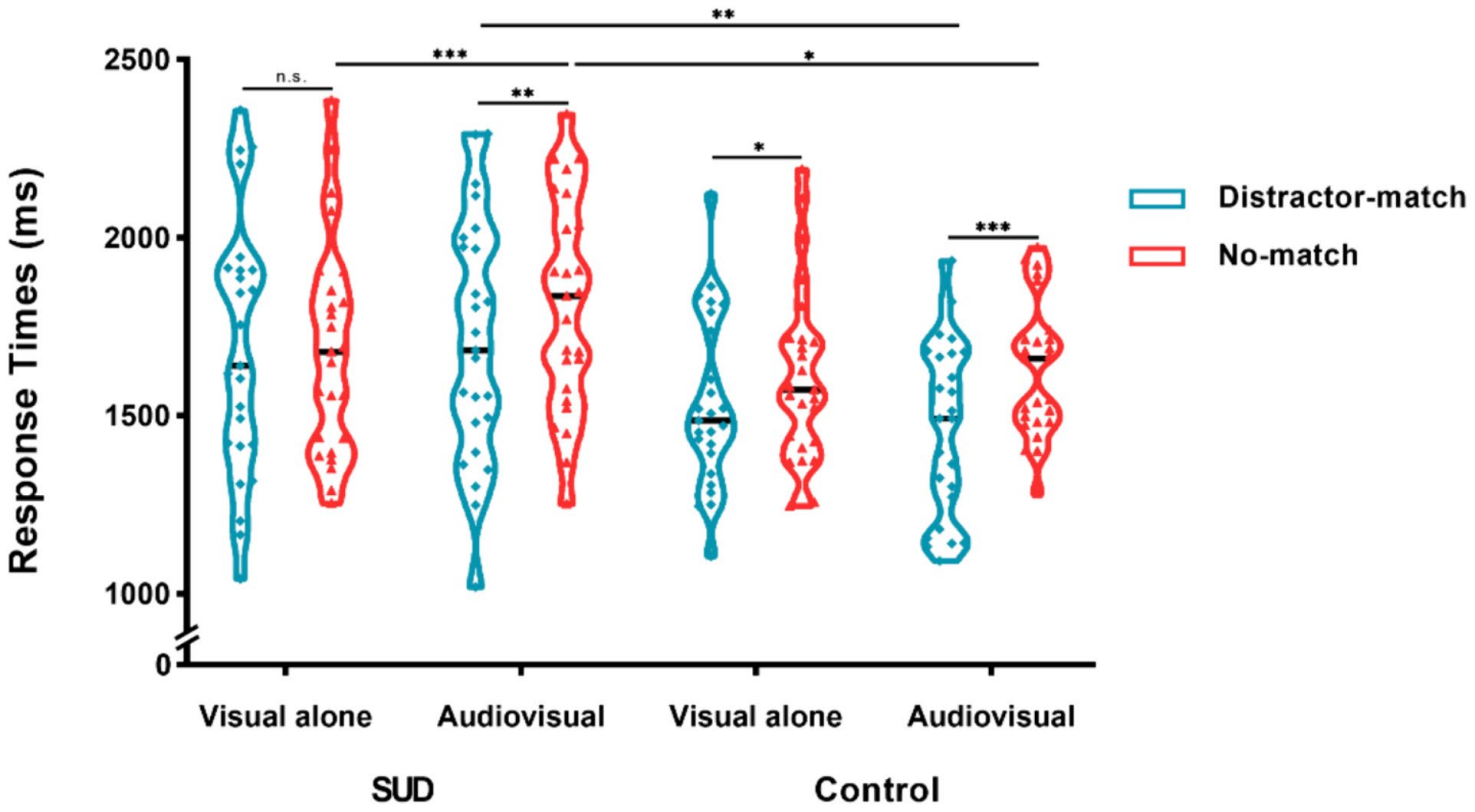
Mean search RTs, shown as a function of the experimental conditions in the SUD group and control group. The black horizontal line indicates the median under this condition. Distractor-match refers to a match between the color represented by the memory sample and the color of one of the distractors in the search display; no-match refers to a mismatch between the memory sample and the search item. Visual alone represents a visual memory sample without auditory information; audiovisual represents a visual memory sample accompanied by auditory congruent information. ^*^p < 0.05, ^**^p < 0.01, and ^***^p < 0.001; n.s., not significant.

For the mean search RTs, the three-way repeated measures ANOVA showed a significant main effect of matching type, *F*(1, 48) = 20.30, *p* < 0.001, η_p_^2^ = 0.30, revealing that search RTs were significantly longer for no-match trials (1,686 ms) than for distractor-match trials (1,599 ms). That is, there was a memory-guided distractor suppression effect. The main effect of group was also significant, *F*(1, 48) = 4.85, *p* = 0.03, η_p_^2^ = 0.09, suggesting that search RTs were longer in the SUD group (1725 ms) than in the control group (1,560 ms). There was also significant two-way interaction between stimuli presentation type and matching type, *F*(1, 48) = 11.31, *p* = 0.002, η_p_^2^ = 0.19, and between group and stimuli presentation type, *F*(1, 48) = 8.20, *p* = 0.006, η_p_^2^ = 0.15. However, the main effect of stimuli presentation type and other interaction effects were not significant, *p*s > 0.05 (Figure 2).

We carried out one-sample *t*-tests for the memory-guided distractor suppression effect (RT_no-match_ – RT_distractor-match_) in the SUD and the control group in each stimuli presentation type, respectively. The results revealed that under the visual alone condition, the suppression effect was significantly greater than 0 in the control group, but not significantly different from 0 in the SUD group, indicating a suppression effect occurred in the control group (*t*(24) = 2.63, *p* = 0.015, *Cohen’s d* = 0.53) rather than in the SUD group (*t* < 1) under the visual alone condition. On the other hand, the suppression effect was markedly greater than 0 in both groups under the audiovisual condition (SUD: *t*(24) = 3.45, *p* = 0.002, *Cohen’s d* = 0.69; Control: *t*(24) = 5.06, *p* < 0.001, *Cohen’s d* = 1.01), indicating a suppression effect occurred in both groups under the audiovisual condition.

Based on the distractor suppression effects in each condition, we discovered that stimuli presentation type modulated the magnitude of the distractor suppression effect in the control group, with greater suppression effects under the audiovisual condition (164 ms) than under the visual alone condition (79 ms), *t*(24) = 2.28, *p* = 0.032, *Cohen’s d* = 0.46. Moreover, between-group comparisons indicated that the distractor suppression effect tended to be greater in the control group (164 ms) compared to the SUD group (105 ms) under the audiovisual condition, but this disparity did not reach statistical significance, *t*(24) = 1.33, *p* > 0.05. This suggested that stimuli presentation type determined whether memory-driven attentional suppression effects occurred in the SUD group; in other words, the SUD group produced attentional suppression effects only under the audiovisual condition. On the other hand, the control group exhibited stable attentional suppression effects, and the effect was boosted by the congruent auditory memory content.

### The time course of memory-guided distractor suppression effect

**Figure 3:**
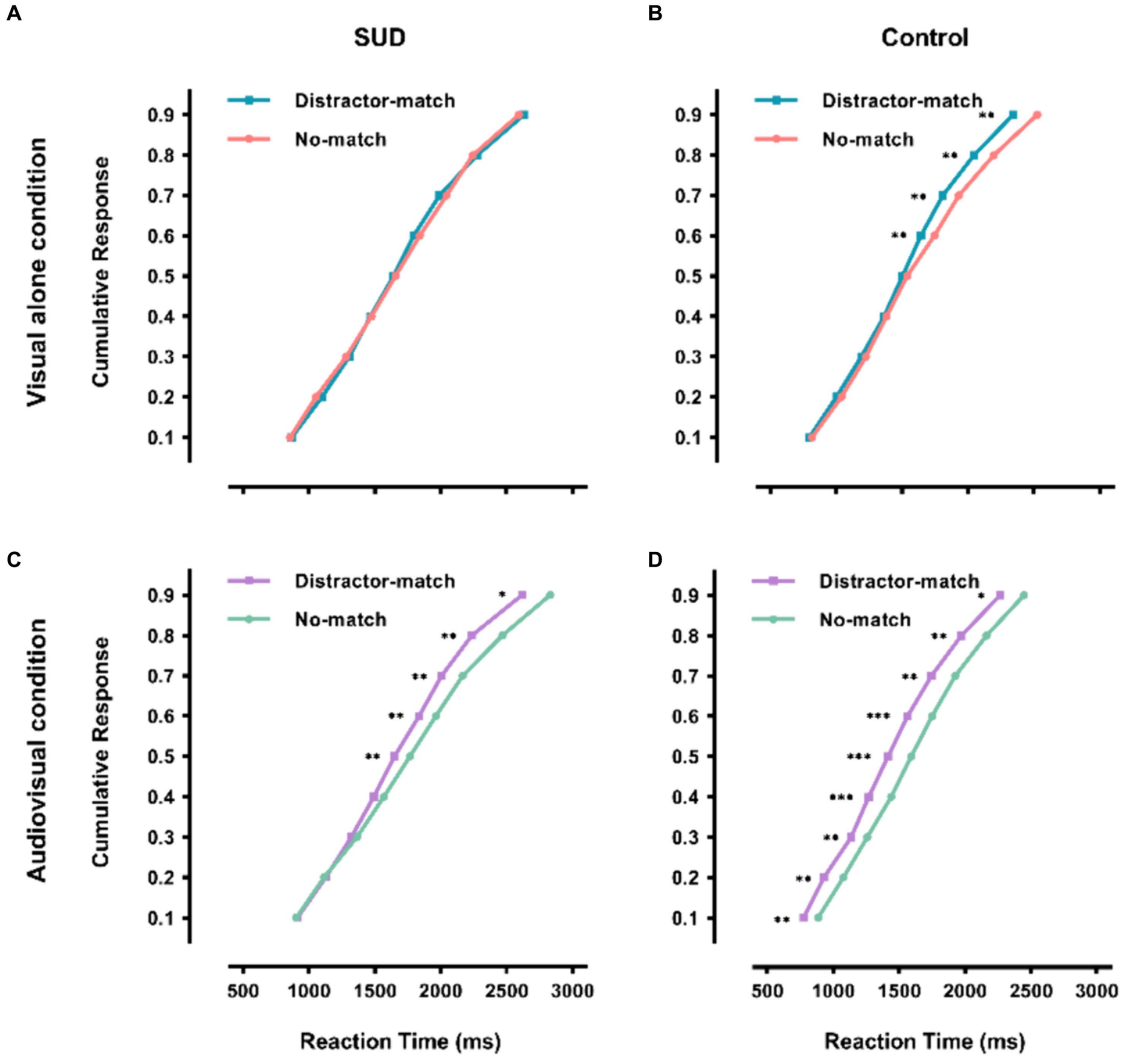
Cumulative probability distribution of search RTs per matching type. Row 1 shows the cumulative probability distribution of the SUD group **(A)** and the control group **(B)** under the visual alone condition. Row 2 shows the cumulative probability distribution of the SUD group **(C)** and the control group **(D)** under the audiovisual condition. Data points represent vincentized values averaging the responses of all subjects in a condition at each quantile. The conditions distractor-match and no-match are the same as those described in Figure 2. ^*^p < 0.05, ^**^p < 0.01, ^***^p < 0.001.

We also investigated whether the congruent auditory memory content affected the time course of memory-guided distractor suppression effect. To do this, we divided the search RT in each condition into ten discrete bins on the basis of cumulative probability RT distribution and then analyzed the difference between no-match and distractor-match conditions at each percentile of RT distribution using Wilcoxon rank order tests (Carlisle and Woodman, 2011b; Cai et al., 2024a,b). As shown in Figure 3, under the visual alone condition, the analyses revealed that search RTs were longer on no-match trials than on distractor-match trials from the 6th to the 9th percentiles in the control group (*p*s < 0.007), but the RT advantage on distractor-match trials was consistently not observed across the percentiles in the RT distribution in the SUD group (*p*s > 0.05). Under the audiovisual condition, the results showed that even in the first percentile of RT distribution, search RTs were substantially longer on no-match trials than on distractor-match trials in the control group (*p*s < 0.032), whereas the difference failed to reach significance until the 5th percentiles in the SUD group (*p*s < 0.025). Additionally, the SUD group was found to have a smaller suppression effect at the 8th and 9th percentile (*p* < 0.006) under the visual alone condition, while this phenomenon only occurred at 1th and 2th percentile (*p* < 0.023) under the audiovisual condition (see Supplementary Figure S1). These results suggested that individuals with SUD, unlike healthy adults, demonstrated no distractor suppression effect driven by visual working memory. Moreover, although congruent auditory memory content could advance the time for cognitive control to engage in attentional guidance, individuals with SUD required additional time to perform as well as healthy adults.

### Relationship between memory-guided distractor suppression effect and self-report scores

The association between self-report scores on the three scales (i.e., RTQ, BSCS, and STAI) and memory-guided distractor suppression effect in each stimuli presentation type (i.e., visual alone and audiovisual conditions) was examined using Pearson’s correlations. Table 1 displays the detailed results.

**Table 1:** Correlations between RTQ, BSCS, STAI, and memory-guided attentional suppression effect.

Measure	M ± SD	Visual alone	Audiovisual	RTQ	BSCS	STAI-state	STAI-trait
Visual alone		−					
Audiovisual		0.34	−				
RTQ		−0.29	−0.30	−			
BSCS		0.07	**0.42** ^*^	**−0.47** ^*^	−		
STAI-state		−0.13	−0.06	**0.52** ^**^	**−0.47** ^*^	−	
STAI-trait		−0.22	0.05	**0.52** ^**^	−0.31	**0.94** ^***^	−

Visual alone refers to the attentional suppression effect under the visual alone condition, audiovisual refers to the attentional suppression effect under the audiovisual condition; RTQ refers to the Relapse Tendency Questionnaire, BSCS refers to Brief Self-Control Scale, STAI-state refers to State Anxiety Subscale, STAI-trait refers to Trait Anxiety Subscale. Bold values represent significant correlation. ^*^p < 0.05, ^**^p < 0.01, ^***^p < 0.001.

Results showed that memory-guided distractor suppression effects were associated with self-control scores. More specifically, under the audiovisual condition, greater distractor suppression effects were related to higher levels of self-control (*r* = 0.42, *p* = 0.035), but not under the visual alone condition (*r* = 0.07, *p* > 0.05). No other self-report scores were significantly associated with memory-driven attentional suppression effects (*p*s > 0.05). Additionally, we found an association between self-control, state anxiety, and relapse tendency, where anxiety and relapse tendency (*r* = 0.52, *p* = 0.008) were positively correlated and self-control was negatively correlated with state anxiety (*r* = −0.47, *p* = 0.017) and relapse tendency (*r* = −0.47, *p* = 0.018).

## Discussion

The present study examined whether there are differences in memory-driven attention between individuals with and without SUD under different stimuli presentation types, and the potential auditory facilitation of memory-driven attention. In the current study, a working memory/visual search dual-task paradigm was used, in which a congruent auditory stimulus was presented in synchronization with a visual working memory item on half of the trials. The results showed there was a memory-guided distractor suppression effect under the visual alone condition, as evidenced by the reliable RT advantage on distractor-match trials. However, this pattern of results was found only in the control group but not in the SUD group. Moreover, both groups exhibited a robust and comparable distractor suppression effect under the audiovisual condition, with a later onset of the suppression effect in the SUD group than in the control group. These results indicated that substance users had a decreased ability to filter interference at the memory level, which could be ameliorated by the congruent auditory memory content.

A memory-guided distractor suppression effect was observed under the visual alone condition in the control group, which is in alignment with the idea that attention to memory-matching distractors could be strategically suppressed by a separate cognitive control mechanism (Woodman and Luck, 2007; Lu et al., 2017). Furthermore, it was found that the SUD group showed neither attentional capture nor attentional suppression across each percentile of the RT distribution. There are two possible interpretations of the current results. One is that individuals with SUD have poor cognitive control ability to effectively utilize working memory representations to modulate attentional allocation and then resist distractor interference from working memory. Alternatively, the absence of attentional guidance effects may simply reflect the lack of any bias signal due to a reduced capacity to apply the controlled strategy in the SUD group. Although both explanations could explain the absence of attentional guidance effects driven by visual working memory in the SUD group, we find the former explanation more convincing given the following evidence. On the one hand, as suggested by both the classical theories (Beck and Kastner, 2009; Han, 2015) and the recent neurophysiological evidence (Carlisle and Woodman, 2011a; Hu and Zhang, 2016), attention is automatically biased toward working memory content, but there is a separate top-down cognitive control mechanism that can override this process. A recent ERP study revealed that memory-guided distractor suppression was affected by the level of cognitive control, but this controlled modulation did not completely eliminate the attentional capture effect, as shown by the fact that memory-matching distractors were still capable of inducing attentional capture-related N2pc at the early phase of visual search (Hu and Zhang, 2016). Moreover, the current study presented a cue display prior to the search display, foreshadowing information about the placement and color of search items. This experimental setting encouraged participants to utilize working memory representation and provided strong motivation to direct attention away from distractors that match the memory content (Han and Kim, 2009).

On the other hand, several prominent explanatory theories of substance addiction, including the self-control failure model and the triadic neurocognitive theory, have highlighted the connection between SUD and diminished executive system control (Baumeister and Vohs, 2016; Turel and Bechara, 2016). Consistent with these theoretical perspectives, previous studies have shown that there is a strong link between SUD and the processing efficiency of the inhibition functions (Smith et al., 2014; Spechler et al., 2016). Research with heroin addicts found that these individuals had lower perceptual sensitivity in a stop-signal task (Ceceli et al., 2023). fMRI results further showed that activity in cognitive control-related regions was negatively correlated with task performance and heroin use severity. The authors interpreted this as indicating that dysregulation of prefrontal cortex-mediated cognitive control results in those with heroin addiction having more difficulty controlling their impulsive behaviors. Additionally, evidence has shown that negative affect is common among individuals with SUD and is increasingly recognized as a part of the phenomenology of addictions (Koob and Volkow, 2016). A few studies have demonstrated that facets of negative affect (e.g., anxiety) have been strongly linked with diminished cognitive control, particularly in filtering interference by irrelevant distractors at the memory level (Luo Y et al., 2021; Salahub and Emrich, 2021). Therefore, negative affect tightly linked to SUD may interfere with the top-down cognitive control system. Correlation analyses provided indirect evidence for this inference; higher state anxiety was associated with a lower level of self-control, which in turn is related to inefficient distractor suppression under the audiovisual condition. Based on these findings, we hold the view that diminished cognitive control in patients with SUD leads to a failure to suppress memory-matching distractors.

It is noteworthy that both groups demonstrated a more pronounced memory-guided distractor suppression effect for audiovisual combinations in comparison to visual alone conditions. Furthermore, the magnitude of this effect was comparable between the two groups. These results might suggest congruent auditory stimuli presented in synchronization with visual working memory content enhanced cognitive control, which in turn enabled individuals with and without SUD to selectively engage attention and suppress memory-matching distractors. The signal suppression hypothesis, which postulated that the relative strengths of inhibitory signals generated by the cognitive control system and priority signals elicited by the stimulus determined the allocation of attention, might account for the current results (Sawaki and Luck, 2011). When the inhibitory signal was stronger than the priority signal, individuals were able to actively suppress the bottom-up response to task-irrelevant distractors (Sawaki et al., 2012). Previous research has shown that audiovisual stimuli may help make up for substance users’ poor attentional control recruitment by engaging brain areas linked to cognitive control and recruiting multimodal attentional resources (Wilcox et al., 2015; Mayer et al., 2017). Additionally, it has been shown that audiovisual memory representations and top-down cognitive control mechanisms can produce a synergistic effect on modulating distractor suppression in visual search (Cai et al., 2024a,b). It is, therefore, plausible to attribute the auditory enhancement of memory-guided distractor suppression to a mechanism in which audiovisual memory representations are integrated with top-down cognitive control demand by task instruction to enhance the strength of the inhibitory signal and optimize the efficiency of visual search. Future work can further address to which the auditory facilitation of memory-guided distractor suppression might be generalized or specific to certain conditions (e.g., does this benefit persist in drug-cue conditions).

Furthermore, the results of the time course of the memory-guided distractor suppression effect showed that a reliable RT advantage on distractor-match trials was not observed until the trials of the 5th percentile under the audiovisual condition in the SUD group. Consistent with this result, some evidence has shown that cognitive control requires sufficient processing time to reconfigure cognitive resources and form a “template for rejection” (Han and Kim, 2009). For instance, studies with healthy adults found that the visual working memory-guided distractor suppression effect occurred relatively late in visual search (Lu et al., 2017; Cai et al., 2024b). Given the effects observed in the SUD group under both visual alone and audiovisual memory encoding conditions, the current results suggested that the congruent auditory memory content could contribute to restoring cognitive control, which leads to an attentional suppression effect in individuals with SUD on trials with sufficient processing time (i.e., a relatively late visual search phase). Similarly, the current results indicated that the auditory facilitation of visual search in the control group. Specifically, the earliest percentile of RT distribution under the audiovisual condition emerged a substantial suppression effect, suggesting that congruent audiovisual memory encoding promoted the time for cognitive control to engage in attentional guidance. These outcomes might be the result of increased cognitive control that is accessible under the audiovisual condition (Cai et al., 2024a,b). Moreover, it should be noted that although memory-guided distractor suppression effects were modulated by the congruent auditory information in both groups, the SUD group exhibited bimodal memory-guided distractor suppression effects later than the control group, and the group differences in the suppression effect could be observed at the 1th and 2th percentile of RT distribution. These results indicate that individuals with SUD may require more effort and rely on greater consumption of cognitive resources to accomplish cognitive control and ultimately perform as well as healthy adults (Mayer et al., 2013).

Regarding the memory performance, in contrast to prior findings showing worse visual working memory performance in the SUD group than the control group (Berenbaum et al., 2023), we did not find any impairment in working memory performance in the SUD group regardless of the modality of the memorandum. This may be ascribed to the fact that there was only one memory load in the delayed matching-to-sample task used in the current study, and this task was relatively simple and therefore well performed by all participants (Zhang et al., 2020). Interestingly, both the SUD group and the control group showed superior performance on the working memory task for audiovisual combinations relative to visual alone conditions, suggesting the auditory facilitation of working memory. Previous multisensory evidence has shown that initially processed visual and auditory information can be integrated by the central executive system into a coherent multisensory representation, hence facilitating cognitive processing (Xie et al., 2017). That is, congruent audiovisual stimulus presentation could improve subsequent memory performance compared with unisensory stimuli through efficient memory encoding (Heikkilä et al., 2015; Yu et al., 2021). The current study expands previous multisensory memory research by demonstrating that SUD patients exhibit audiovisual advantages, as well as healthy adults, in a working memory/attention dual-task paradigm with verbal stimuli as memorandums. Thus, the current study adds to the body of evidence supporting the integrated perception-cognition theory (Schneider and Pichora-Fuller, 2000; Cai et al., 2024a,b), suggesting that congruent auditory information can facilitate perception processing, resulting in leaving more cognitive resources available for later cognitive processes, such as working memory and memory-guided distractor suppression.

The present findings not only contribute to a more profound comprehension of the interrelationship existing between working memory, cognitive control, and attentional guidance in SUD, but also provide important clinical implications for diagnosis and intervention for SUD. Our findings suggest that memory guidance in distractor suppression can serve as a promising transdiagnostic cognitive risk marker for SUD, yet more work is needed to verify this idea. Moreover, the relationship between anxiety, self-control, and relapse tendency indicates that rehabilitation centers can help addicts reduce relapse by improving their emotional regulation and self-control abilities. Furthermore, evidence has shown that attentional bias correction can be effective in reducing relapse of individuals with SUD (Field et al., 2014). Previous experimental paradigms most commonly used for attentional bias modification in substance users (such as the dot-probe paradigm, cue-target paradigm, and simple visual search task) have typically examined and trained attentional functions in a de-working memory context that does not close to actual social situations (Cox et al., 2014; Heitmann et al., 2018). Thus, memory-driven attentional tasks can be adapted to a targeted intervention measure for SUD. However, note that the experimental materials selected for the current study were general visual and auditory stimuli (color and its written and spoken word) rather than specific addictive stimuli; this allows us to investigate whether substance addiction is associated with a general deficit of distractor suppression related to non-drug stimuli. Previous studies have shown that substance-related stimuli induce high reward salience signals that capture the attention of individuals with SUD (Robinson and Berridge, 1993; Metrik et al., 2016) and that reward salience can synergize cognitive control to modulate the allocation of attention and facilitate memory-guided distractor suppression (Gong et al., 2016). Combined with the present findings, it can be speculated that individuals with SUD may flexibly adjust attentional allocation in the context of drug cues to inhibit substance-related distractors. Therefore, memory-driven attention tasks can be designed in the context of multisensory substance-related stimuli and used to improve the ability of illicit drug users to utilize working memory to filter interference and reduce the relapse of individuals with SUD.

The current study has several limitations that need to be addressed. First, while cognitive control deficits are commonly observed across SUDs (Zilverstand et al., 2018), research has revealed that people who use distinct types of drugs exhibit variations in different tasks measuring cognitive control, including working memory, inhibitory control, and cognitive flexibility (Badiani et al., 2011; Smith et al., 2014). Therefore, future research needs to further analyze the potential effects of drug types on memory guidance in distractor suppression. Second, this study left unresolved which specific inhibitory mechanism (reactive or proactive inhibition) is involved in these behavioral effects, and follow-up research needs to use electroencephalography and neuroimaging techniques to further investigate this issue. Third, there is evidence that females and males with SUD differ in alterations in brain regions related to cognitive control and behavioral measures of inhibitory control (Weafer and de Wit, 2014; Kogachi et al., 2017). Future research needs to further explore the importance of sex when examining resistance to proactive interference at the memory level.

Despite these limitations, the current results indicate that individuals with SUD are not capable of resisting proactive interference at the memory level, possibly due to cognitive control deficits. But when provided with congruent auditory information during the working memory encoding phase, the deficit in distractor suppression can be ameliorated. This is demonstrated by the fact that individuals with SUD can take advantage of congruent audiovisual memorandum to filter interference by memory-matching distractors, but this requires a longer period of time to exhibit similar levels of memory-guided distractor suppression as healthy adults. Moreover, greater distractor suppression driven by audiovisual memory is related to weaker substance addiction.

## Data availability statement

The raw data supporting the conclusions of this article will be made available by the authors, without undue reservation.

## Ethics statement

The studies involving humans were approved by the Institutional Review Board of the Academic Committee of the Department of Psychology, Soochow University, China (protocol code SUDA20220714H02). The studies were conducted in accordance with the local legislation and institutional requirements. The participants provided their written informed consent to participate in this study.

## Author contributions

BC: Conceptualization, Formal analysis, Investigation, Software, Writing – original draft. JW: Formal analysis, Visualization, Writing – original draft. HS: Funding acquisition, Methodology, Writing – review & editing. ZZ: Conceptualization, Funding acquisition, Validation, Writing – review & editing. AW: Conceptualization, Funding acquisition, Methodology, Resources, Writing – review & editing.
