# Supplementary material for: The auditory stimulus facilitates memory guidance in distractor suppression in males with substance use disorder
Source: Front Psychol. 2024 Jul 3;15:1417557. doi: 10.3389/fpsyg.2024.1417557 (PMC11259375; doi:10.3389/fpsyg.2024.1417557)
Supplement: Supplementary file 1 [file Table_1.DOCX]

**Table S1** Demographic and drug abuse-related characteristics of the SUD and control group.

| **Condition** |  | **SUD (n = 25)** | **Control (25)** |  |
| --- | --- | --- | --- | --- |
| Age (years) |  | 31.80 ± 4.90 | 30.92 ± 4.43 |  |
| Education Level (N/%) | Primary school or below | 3 (12%) | 1 (4%) |  |
|  | Secondary school | 16 (64%) | 11 (44%) |  |
|  | High school | 4 (16%) | 7 (28%) |  |
|  | College and above | 2 (8%) | 6 (24%) |  |
| Occupation (N/%) | Unemployed | 7 (28%) | 3 (12%) |  |
|  | Employed | 9 (36%) | 14 (56%) |  |
|  | Self-employed | 9 (36%) | 8 (32%) |  |
| Family status (N/%) | Family integrity | 16 (64%) | 18 (72%) |  |
|  | Single-parent family (father) | 6 (24%) | 3 (12%) |  |
|  | Single-parent family (mother) | 3 (12%) | 4 (16%) |  |
| Marital status (N/%) | Unmarried | 17 (68%) | 15 (60%) |  |
|  | Married | 8 (32%) | 10 (40%) |  |
| Children’s status (N/%) | None | 14 (56%) | 17 (68%) |  |
|  | One | 5 (20%) | 6 (24%) |  |
|  | More than one | 6 (24%) | 2 (8%) |  |
| Types of drug use (N/%) | Traditional drug | 6 (24%) | — |  |
|  | New drug | 19 (76%) |  |  |
| Number of detoxifications (N/%) | One | 17 (68%) | — |  |
|  | More than one | 8 (32%) |  |  |
| Years of drug use (N/%) | 0-5 years | 7 (28%) | — |  |
|  | 6-10 years | 6 (16%) |  |  |
|  | 11-15 years | 11 (44%) |  |  |
|  | 16-20 years | 1 (4%) |  |  |

*Note.* Based on the chronology of drug prevalence, drug types were divided into two categories, traditional durgs (heroin, cocaine, and cannabis) and new drugs (methamphetamin and ecstasy)

**Table S2** Mean memory and search CRs as well as search RTs (with standard errors) in each condition.

| Group | Stimuli presentation type | Matching type | Search RT (ms) | Search CR (%) | Memory CR (%) |
| --- | --- | --- | --- | --- | --- |
| SUD | Visual alone | Distractor-match | 1690 (73) | 96.1 (1.1) | 95.1 (1.1) |
|  |  | No-match | 1690 (60) | 96.2 (1.4) | 95.1 (1.4) |
|  | Audiovisual | Distractor-match | 1707 (68) | 97.9 (0.8) | 98.8 (0.3) |
|  |  | No-match | 1812 (60) | 96.8 (0.8) | 98.3 (0.4) |
| Control | Visual alone | Distractor-match | 1533 (48) | 96.8 (1.2) | 96.2 (0.8) |
|  |  | No-match | 1612 (49) | 96.9 (0.7) | 95.9 (0.8) |
|  | Audiovisual | Distractor-match | 1466 (49) | 96.1 (1.0) | 98.9 (0.4) |
|  |  | No-match | 1630 (38) | 96.2 (0.8) | 98.3 (0.5) |


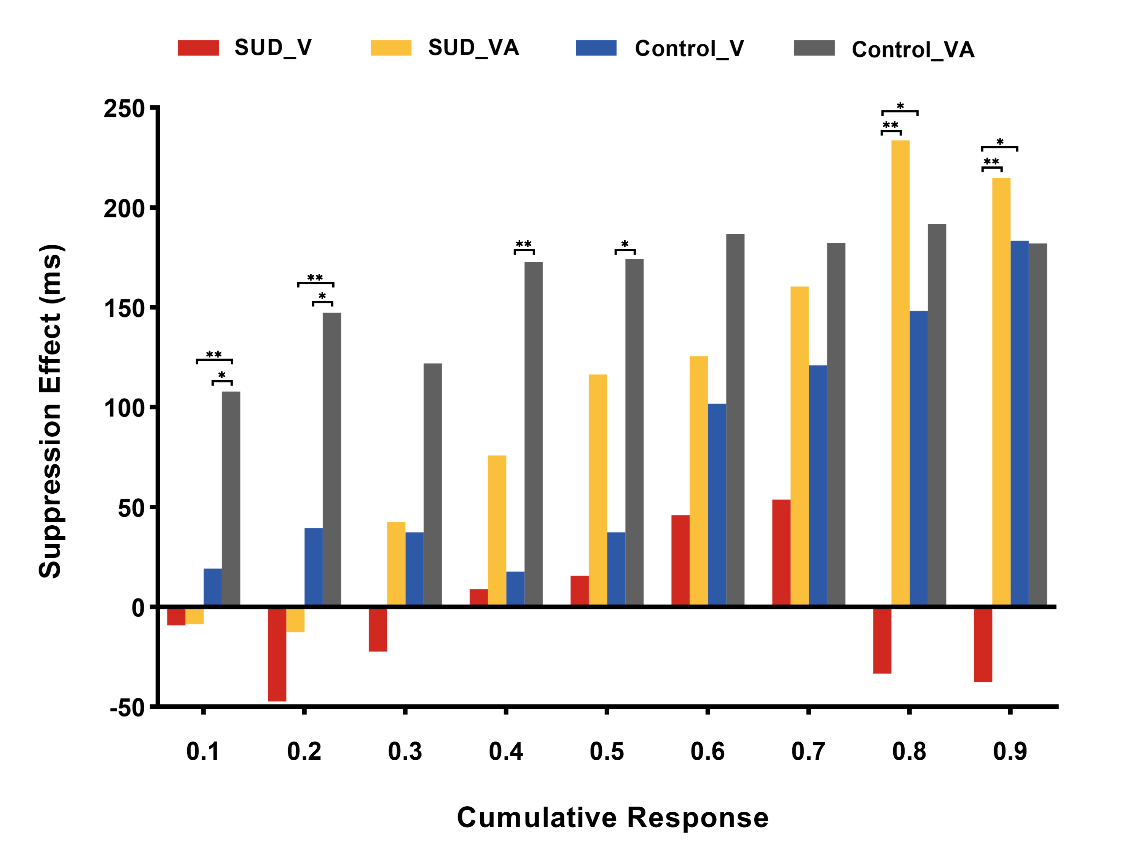


**Figure S1** Memory-guided suppression effect as a function of stimuli presentation type in the SUD group and control group at each quantile of RT distribution. SUD_V and SUD_VA refer to the suppression effect under the visual alone and the audiovisual condition in the SUD group, respectively; Control_V and Control_VA refer to the suppression effect under the visual alone and the audiovisual condition in the Control group, respectively. **p* < 0.05, ***p* < 0.01, and ****p* < 0.001.
